# Comprehensive analysis of the expression and significance of CXCLs in human diffuse large B-cell lymphoma

**DOI:** 10.1038/s41598-022-06877-2

**Published:** 2022-02-18

**Authors:** Xiaonan Zhou, Shizhu Guo, Yonghong Shi

**Affiliations:** 1 School of Basic Medical Sciences, Inner Mongolia Medical University, Hohhot, 010110 Inner Mongolia Autonomous Region China; 2 Department of Pathology, Affiliated Hospital of Inner Mongolia Medical University, Hohhot, Inner Mongolia Autonomous Region China

**Keywords:** Cancer, Computational biology and bioinformatics

## Abstract

CXCL chemokines (CXCLs) are small cytokines or signal proteins secreted by cells that have been proven to be linked to the occurrence and development of many kinds of cancer. However, the expression and diagnostic and prognostic value of CXCLs in diffuse large B-cell lymphoma (DLBCL) remain to be further studied. We obtained CXCL transcription and survival data of patients with DLBCL from Oncomine, Gene Expression Profiling Interactive Analysis (GEPIA), The Cancer Genome Atlas (TCGA), TIMER and cBioPortal databases. R software, STRING and EXCEL were used to process the data. This study discovered that the expression levels of CXCL9-14 in DLBCL were higher than those in normal tissues, while CXCL4, CXCL7 and CXCL8 were lower in tumor than in normal tissues*.* The expression levels of CXCL2, CXCL10 and CXCL11 were related to tumor stage. CXCL9-14 could be used as an auxiliary molecular marker for the diagnosis of DLBCL. CXCL17 might be a potential prognostic marker of DLBCL.

## Introduction

Chemokines are small molecules of cytokines that can induce chemotactic movement of cells. According to the position of the N-terminal cysteine, chemokines are classified into four major subfamilies: CXC, CC, C and CX3C and CXC, CC, C and CX3C. Their corresponding receptors are CXCR, CCR, CR and CX3CR^1^. The CXC chemokine family is divided into ELR chemokines and non-ELR chemokines based on whether there exists a glutamic acid-leucine-arginine (ELR) functional region in front the first cysteine. CXC members induce chemotactic activity and play a vital role in inflammation and immunity, and are the key inflammatory mediators associated with the presence of chronic inflammatory cells in cancer microenvironment. Meanwhile, they are also targets of carcinogenic pathways. They initially play a prominent role in determining the tumor stromal composition and affect the proliferation and metastasis of cancer cells^2^. Many studies have confirmed that most tumors can autonomously secrete CXC chemokines to promote cell proliferation, growth and metastasis as well as angiogenesis in tumor tissues^3^. The expression of CXCLs is out of control in the cancer of liver, breast and esophageal, lymphoma, and other human malignant tumors^4–6^.

Lymphoma is a malignancy of the lymphatic system that can be produced by B lymphocytes, T lymphocytes or natural killer (NK) cells at various stages of maturation. Clinic characteristic is painless progressive lymphadenopathy. That can be produced by B lymphocytes, T lymphocytes or natural killer (NK) cells at various stages of maturation. Clinic characteristic is painless progressive lymphadenopathy. It is divided into Hodgkin's lymphoma (HL) and non Hodgkin's lymphoma (NHL). NHL accounts for 80% of all lymphomas. Diffuse large B-cell lymphoma (DLBCL) is currently recognized the one of the most common forms of NHL^7^.

DLBCL is characterized by diffuse infiltration of medium to large cells with large nucleoli and abundant cytoplasm, which destroys and eliminates the histologic structure of the lymph nodes involved. The tumor cells express B cell markers such as CD19, CD20, CD22, CD45 and CD79. Moreover, CD30 is expressed in approximately 40% of cases and predicts a good prognosis^8,9^. While immunological and clinical features have limited influence on its prognosis, recent study has focused on the molecular factors. Advances in gene expression profiling have shown that DLBCL contains three different molecular subtypes: (i) germinal center B-cell like (GCB-like) type, which expresses the molecular markers of normal germinal center B cells and has a good prognosis; and (ii) activated B-cell-like (ABC-like) type, which expresses molecular markers of activated B cells and plasma cells and usually showed poor prognosis. (iii) Unclassified, it has the same prognosis as ABC without definite molecular characteristics.

The expression of MYC, BCL6 and BCL2 in DLBCL is associated with disease progression. Recently, TP53 mutation has been increasingly recognized as a feature associated with chemotherapy resistance and low survival. However, the current biomarkers for predicting prognosis still have some limitations. New biomarkers need to be discovered to predict as prognosis and to achieve effectively individualized treatment.

To date, 17 CXC factors have been identified in mammalian cells. They are numbered according to the discovery sequence (CXCL1, CXCL2, CXCL3, CXCL4, CXCL5, CXCL6, CXCL7, CXCL8, CXCL9, CXCL10, CXCL11, CXCL12, CXCL13, CXCL14, CXCL15, CXCL16, and CXCL17). CXCL15 was not involved in this study because there was no information about CXCL15 in the database we used. We hold the view that CXCLs have complex and unique roles in DLBCL. They found that in a considerable number of DLBCL patients, tumor cells constitutively produce the chemokine CXCL8, enabling them to recruit APRIL to induce neutrophils. CXCL8 production affects DNA methylation and acetylation. It mediates neutrophil recruitment and secretes the tumor promoter of APRIL-mediated DLBCL progression^10^. Epstein-Barr virus (EBV) encoded microRNAs (miRNAs) targeting CXCL11 with potent antitumor activity and may be the immuno-modulatory mechanism of DLBCL^11^. CXCL12 and CXCL13 mediate chemotaxis of lymphoma cells. CXCL13 serves as a prognostic and diagnostic biomarker for CNS lymphoma^12^. Abnormal CXCL expression and its relationship with clinicopathological features and prognosis have been partially reported in human DLBCL. To our knowledge, bioinformatics analyses have not been applied to explore the role of CXCLs in DLBCL. With the development of science and technology, as well as the advancement of medical technology, DNA and RNA research has gradually become a principal part of biological and biomedical research. By mining thousands of online gene expression or replication data, we analyzed the expression and potential functions of CXCLs in patients with DLBCL under different factors, providing value for the treatment and prognosis of DLBCL.

## Materials and methods

### Oncomine analysis

Oncomine (http://www.oncomine.org)^13^ is a cancer microarray database and integrated data mining platform designed to facilitate discovery from genome-wide expression analysis. We used Oncomine to analyze the transcriptional level of CXCLs in different tumors. The mRNA expression of CXCLs in tumor tissues was compared with that of normal tissues, and the P-value was obtained by t-test. The limits of the P-value and fold change were 0.01 and 2, respectively.

### TCGA dataset

TCGA (The Cancer Genome Atlas) (https://www.cancer.gov/tcga)^14^ is a project corporately launched by the National Cancer Institute (NCI) and the National Human Genome Research Institute (NHGRI) in 2006. It collects clinical data and genomic variation of various human cancers (including subtypes). mRNA expression, miRNA expression, methylation and other data are important data sources for cancer researchers. We used data from the TCGA database DLBC (diffuse large B-cell lymphoma) project and R (version 4.1.1) (Basic R package) to analyze CXCL expression in tumor stages and judged the diagnostic value of CXCLs in patients with DLBCL. P-value < 0.05 were considered significant.

### GEPIA dataset

GEPIA (http://gepia.cancer-pku.cn/)^15^ is a newly developed interactive web server for analyzing the RNA sequencing expression of a large number of tumors and normal samples from the TCGA and GTEx projects using a standard processing pipeline. Moreover, GEPIA provides customizable functions, such as tumor/normal differential expression analysis, patient survival analysis, and similar gene detection. We, to explore the expression of CXCLs in DLBCL and the prognostic value of the mRNA level of CXCLs in DLBCL patients. We also used EXCEL drawing diagrams to show the correlations between CXCLs, statistical method: Spearman, P-value < 0.05 were considered significant.

### cBioPortal

cBioPortal for Cancer Genomics (cBioPortal, https://www.cbioportal.org/)^16,17^ contains data from 126 tumor genomic studies, including large tumor research projects such as TCGA and ICGC, covering 28,000 samples, as well as phenotypic information such as clinical prognosis. We used the cBioPortal online tool to analyze the changes in CXCLs.

### STRING

STRING (https://string-db.org/, version 11.5)^18^ can search online for known protein interactions. We used STRING to build the network for CXCLs and frequently altered neighboring genes.

### R

R is a free software environment for statistical computing and graphics^19^. We used R (version 4.1.1) (http://www.r-project.org) (pROC package GGploT2 package) to analyze the diagnostic value of CXCLs in patients with DLBCL. The closer the AUC is to 1, the better the diagnostic effect is. It has low accuracy when AUC is between 0.5 and 0.7. AUC has a certain accuracy between 0.7 and 0.9. While AUC above 0.9 has high accuracy. We used R (ggplot2 package and clusterprofiler package) to explore the correction between different CXCLs in DLBCL and predicted the functions of CXCLs and the genes significantly related to the alterations of CXCLs by analyzing conduct Kyoto encyclopedia of genes and genomes (KEGG)^20–22^. In the enrichment results, adjusted P < 0.05 was deemed a meaningful pathway.

### TIMER

TIMER (https://cistrome.shinyapps.io/timer/, version 2.0)^23–25^ is a web server for comprehensive analysis of Tumor-Infiltrating Immune Cells. We obtained the relationship between CXCL expression and immune infiltration through TIMER2.0 database. We also used EXCEL drawing diagrams to show the correlations between them, statistical method: Spearman, P-value < 0.05 were considered significant.

### Data availability

The data used to support the findings of this study are openly available from TCGA database. Citing TCGA in Publications and Presentations was originally published by the National Cancer Institute. The results < published or shown > here are in whole or partly based upon data generated by the TCGA Research Network : https://www.cancer.gov/tcga.

### Consent for publication

All authors agreed to publish.

## Results

### Transcription levels of CXCLs in patients with DLBCL

**Figure 1:**
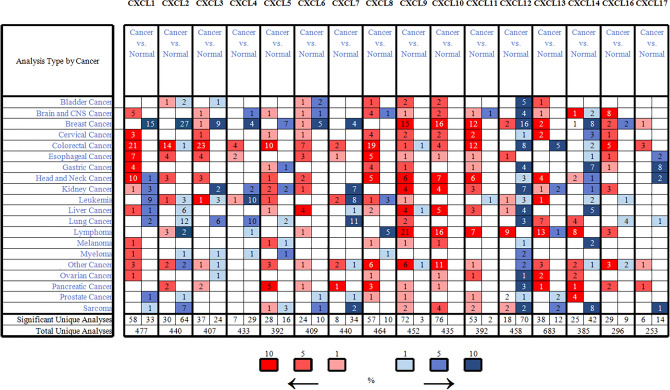
The Transcription Levels of CXCLs in different types of cancers (Oncomine database, http://www.oncomine.org) (note: red indicates the high expression of CXCLs, blue indicates the low expression of CXCLs, and number indicates the number of CXCLs expression cases).

Seventeen CXCL factors were found in mammalian cells. We used the Oncomine database to compare CXCL transcription levels in a variety of cancer samples with normal samples. CXCL9, CXCL10, CXCL11, CXC12, CXCL13, and CXCL14 expression in the tissue adjacent to carcinoma was significantly different (Fig. 1). We also summarized the transcription of 16 chemokine families in DLBCL from eight databases. The changes in the mRNA expression levels of CXCL1, CXCL3-8 and CXCL17 in lymphoma patients were not particularly significant (P > 0.05) (Table 1). Next, we focused on the expression of CXCLs in samples of DLBCL. In Compagno Lymphoma Statistics^26^, CXCL2 was overexpressed in DLBCL versus normal tissue with a fold change of 2.544 consistent with Alizadeh Lymphoma Statistics^27^**.** In Alizadeh Lymphoma Statistics^27^, CXCL9 was found to be more highly expressed in DLBCL supported by Storz^28^, Rosenwald^29^, Compagno^26^, Brune^30^, and Basso^31^ (Table 1). Rosenwald multicancer statistics^29^ showed that CXCL10 was increased in DLBCL (fold change = 5.390) compared to normal samples. The same results were reported by Compagno, Basso^31^, and Storz^28^ (Table 1). In Basso’s dataset^31^ and Compagno’s dataset^26^, CXCL11 had a significant increase in DLBCL. CXCL12 was also highly expressed in DLBCL than in normal samples^26–28^. Alizadeh’s dataset^27^, Basso’s dataset^31^, and Compagno’s dataset^26^ all indicated that CXCL13 was more highly expressed in DLBCL (Table 1). In Compagno Lymphoma Statistics^26^, CXCL14 was overexpressed in DLBCL versus normal tissue with a fold change of 5.168. CXCL16 was overexpressed with a fold change of 3.107 (Table 1).

**Table 1:** Significant changes in CXCL expression at the transcription level between DLBCL and normal tissues (Oncomine DATABASE).

	Fold change	P-value	t-test	Source and/or reference
CXCL2	2.544	1.73E−6	5.101	Compagno lymphoma statistics
	3.005	0.015	3.853	Alizadeh lymphoma statistics
CXCL9	9.957	0.002	5.659	Alizadeh lymphoma statistics
	24.803	6.37E−4	5.642	Storz lymphoma statistics
	17.389	3.86E−16	14.862	Rosenwald multicancer statistics
	19.907	1.44E−8	7.017	Basso lymphoma statistics
	54.026	6.12E−31	23.643	Compagno lymphoma statistics
	19.134	3.27E−5	6.522	Brune lymphoma statistics
CXCL10	5.390	8.61E−9	7.348	Rosenwald multicancer statistics
	8.586	9.08E−7	5.484	Basso lymphoma statistics
	22.362	8.76E−27	19.120	Compagno lymphoma statistics
	7.699	0.001	5.144	Storz lymphoma statistics
CXCL11	14.345	1.38E−19	15.464	Compagno lymphoma statistics
	15.820	3.09E−8	6.825	Basso lymphoma statistics
CXCL12	2.438	1.03E−5	5.044	Alizadeh lymphoma statistics
	3.254	1.66E−6	6.258	Rosenwald multicancer statistics
	3.485	1.74E−15	11.315	Compagno lymphoma statistics
CXCL13	10.710	0.024	3.108	Alizadeh lymphoma statistics
	10.064	4.36E−9	6.802	Basso lymphoma statistics
	17.816	1.09E−12	9.726	Compagno lymphoma statistics
CXCL14	5.168	2.59E−11	8.320	Compagno lymphoma statistics
CXCL16	3.107	2.39E−18	12.583	Compagno lymphoma statistics

### Relationship between CXCL mRNA levels and clinicopathological parameters in patients with DLBCL

**Figure 2:**
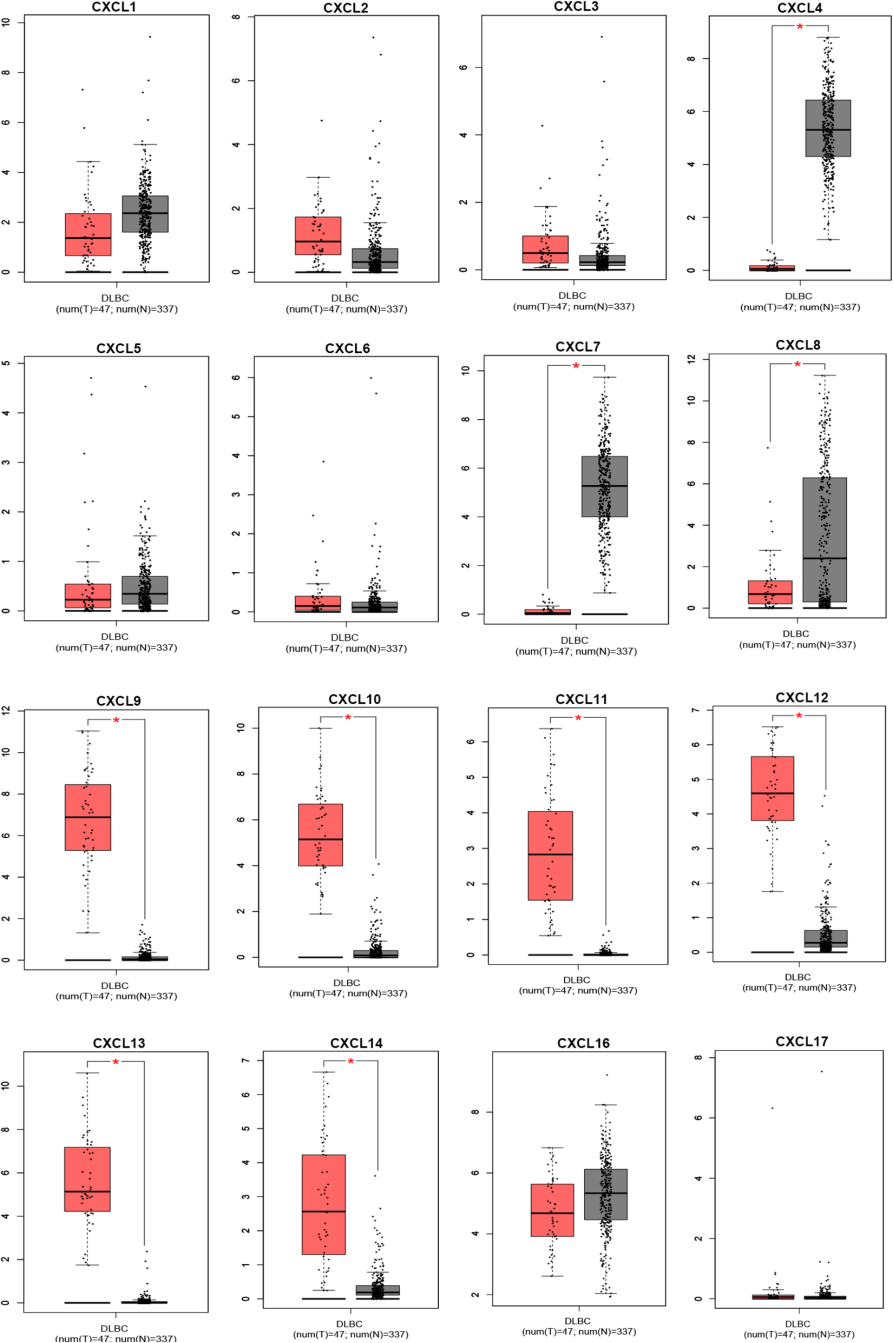
Expression of CXCLs in DLBCL (GEPIA, http://gepia.cancer-pku.cn/). The expression of CXCLs in DLBCL (box plot) (note: red represents tumor tissue and gray represents normal tissue).

Using the GEPIA (Interactive Analysis of Gene Expression Profiles) dataset (http://gepia.cancer-pku.cn/), we compared the mRNA expression of CXCL factors in DLBCL with normal tissue. The findings revealed that the expression levels of CXCL9, CXCL10, CXCL11, CXCL12, CXCL13, CXCL14 were higher than normal tissue, the expression levels of CXCL4,CXCL7 and CXCL8 were lower than that of normal tissue (P < 0.05) (Fig. 2). We also used data from the TCGA database DLBC project to analyze CXCL expression in DLBCL. The results indicated that the expression of CXCLs was related to tumor stage. There was a significant relationship with tumor stage in the group of CXCL2, CXCL10 and CXCL11 (P < 0.05). In contrast, the expression of CXCL1, CXCL3-9, CXCL12-14 and CXCL16-17 had no significant relationship with tumor stage (Table 2).

**Table 2:** Correlation between CXCL expression and tumor stage in DLBCL (TCGA).

Clinical stage, n (%)		Stage I	Stage II	Stage III	Stage IV	p
**Characteristic**						
CXCL1	High expression	2 (4.8%)	9 (21.4%)	2 (4.8%)	7 (16.7%)	0.544
	Low expression	6(14.3%)	8 (19%)	3 (7.1%)	5 (11.9%)	
**CXCL2**	**High expression**	**0 (0%)**	**10(23.8%)**	**3 (7.1%)**	**7 (16.7%)**	**0.019**
	**Low expression**	**8 (19%)**	**7 (16.7%)**	**2 (4.8%)**	**5 (11.9%)**	
CXCL3	High expression	1 (2.4%)	9 (21.4%)	4 (9.5%)	7 (16.7%)	0.088
	Low expression	7(16.7%)	8 (19%)	1 (2.4%)	5 (11.9%)	
CXCL4	High expression	3 (7.1%)	8 (19%)	3 (7.1%)	6 (14.3%)	0.917
	Low expression	5(11.9%)	9 (21.4%)	2 (4.8%)	6 (14.3%)	
CXCL5	High expression	2 (4.8%)	9 (21.4%)	3 (7.1%)	6 (14.3%)	0.580
	Low expression	6 (14.3%)	8 (19%)	2 (4.8%)	6 (14.3%)	
CXCL6	High expression	3 (7.1%)	9 (21.4%)	2 (4.8%)	6 (14.3%)	0.917
	Low expression	5 (11.9%)	8 (19%)	3 (7.1%)	6 (14.3%)	
CXCL7	High expression	5 (11.9%)	9 (21.4%)	2 (4.8%)	5 (11.9%)	0.845
	Low expression	3 (7.1%)	8 (19%)	3 (7.1%)	7 (16.7%)	
CXCL8	High expression	4 (9.5%)	8 (19%)	4 (9.5%)	4 (9.5%)	0.387
	Low expression	4 (9.5%)	9 (21.4%)	1 (2.4%)	8 (19%)	
CXCL9	High expression	2 (4.8%)	8 (19%)	3 (7.1%)	7 (16.7%)	0.554
	Low expression	6 (14.3%)	9 (21.4%)	2 (4.8%)	5 (11.9%)	
**CXCL10**	**High expression**	**1 (2.4%)**	**7 (16.7%)**	**5 (11.9%)**	**7 (16.7%)**	**0.015**
	**Low expression**	**7 (16.7%)**	**10(23.8%)**	**0 (0%)**	**5 (11.9%)**	
**CXCL11**	**High expression**	**1 (2.4%)**	**8 (19%)**	**5 (11.9%)**	**6 (14.3%)**	**0.021**
	**Low expression**	**7 (16.7%)**	**9 (21.4%)**	**0 (0%)**	**6 (14.3%)**	
CXCL12	High expression	4 (9.5%)	6 (14.3%)	3 (7.1%)	6 (14.3%)	0.742
	Low expression	4 (9.5%)	11 (26.2%)	2 (4.8%)	6 (14.3%)	
CXCL13	High expression	5 (11.9%)	6 (14.3%)	3 (7.1%)	4 (9.5%)	0.485
	Low expression	3 (7.1%)	11 (26.2%)	2 (4.8%)	8 (19%)	
CXCL14	High expression	4 (9.5%)	6 (14.3%)	4 (9.5%)	6 (14.3%)	0.379
	Low expression	4 (9.5%)	11 (26.2%)	1 (2.4%)	6 (14.3%)	
CXCL16	High expression	2 (4.8%)	10 (23.8%)	2 (4.8%)	7 (16.7%)	0.434
	Low expression	6 (14.3%)	7 (16.7%)	3 (7.1%)	5 (11.9%)	
CXCL17	High expression	4 (9.5%)	9 (21.4%)	3 (7.1%)	4 (9.5%)	0.690
	Low expression	4 (9.5%)	8 (19%)	2 (4.8%)	8 (19%)	

Significant values are in bold.

### The diagnostic value of CXCLs in patients with DLBCL

**Figure 3:**
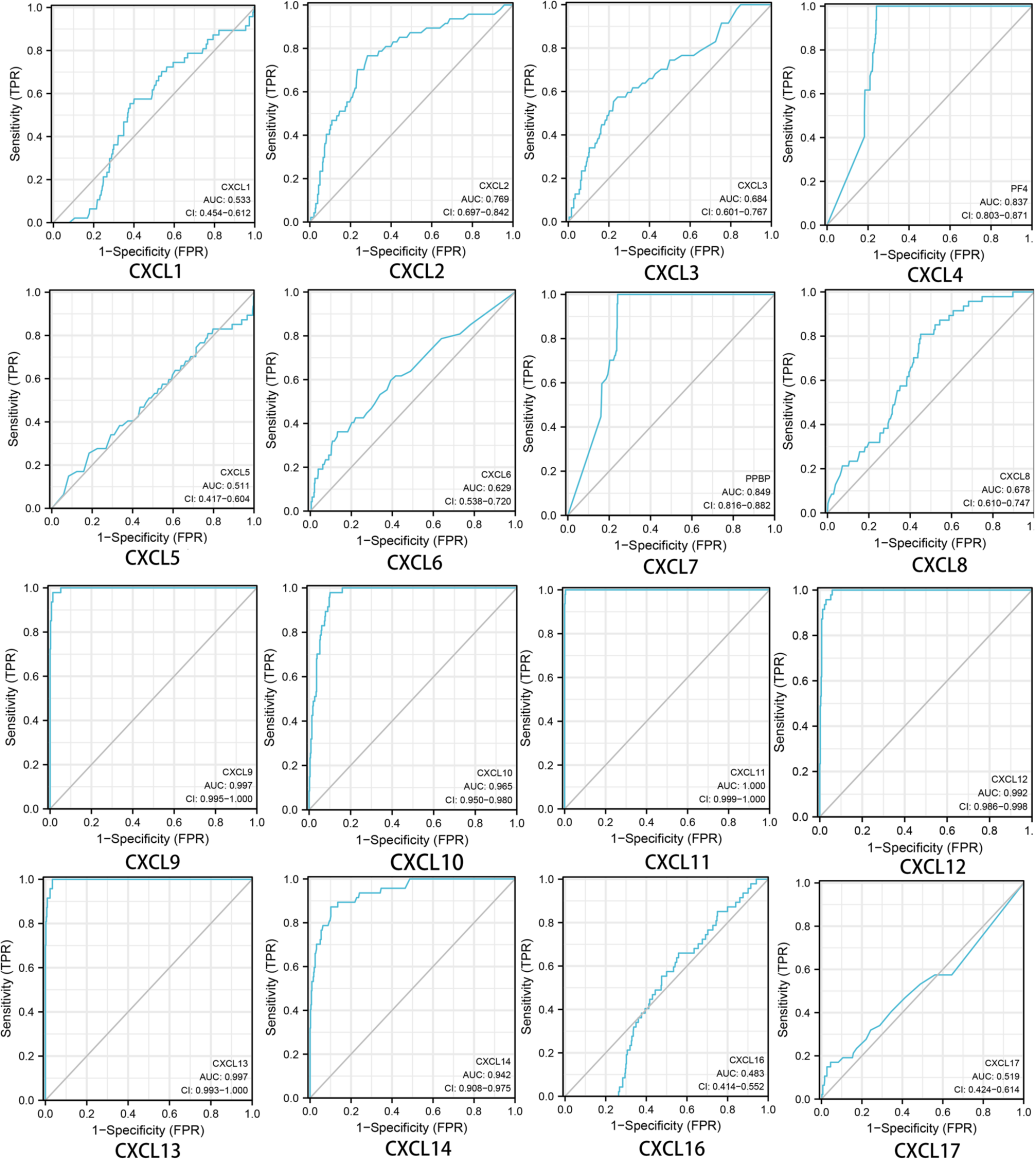
Correlation between CXCL expression and diagnosis in DLBCL (TCGA, R version 4.1.1, http://www.r-project.org).

We further investigated the role of CXCLs in the diagnostic value of patients with DLBCL. We used R (pROC package GGploT2 package) to analyze and visualize the data. The results displayed that the prediction ability was high in CXCL9(AUC = 0.997), CXCL10(AUC = 0.965), CXCL11(AUC = 1.000), CXCL12(AUC = 0.992), CXCL13(AUC = 0.997) and CXCL14(AUC = 0.942) and low in CXCL1(AUC = 0.533), CXCL3 (AUC = 0.684), CXCL5(AUC = 0.511), CXCL6(AUC = 0.629), CXCL8 (AUC = 0.678) and CXCL17(AUC = 0.519), and was a certain degree of accuracy in CXCL2 (AUC = 0.769), CXCL4(AUC = 0.837) and CXCL7(AUC = 0.849). In addition, CXCL16 had poor predictive power and diagnostic efficacy (AUC = 0.483) (shown in Fig. 3).

### Association of the increased or decreased CXCL mRNA expression and improved prognosis in patients with DLBCL

**Figure 4:**
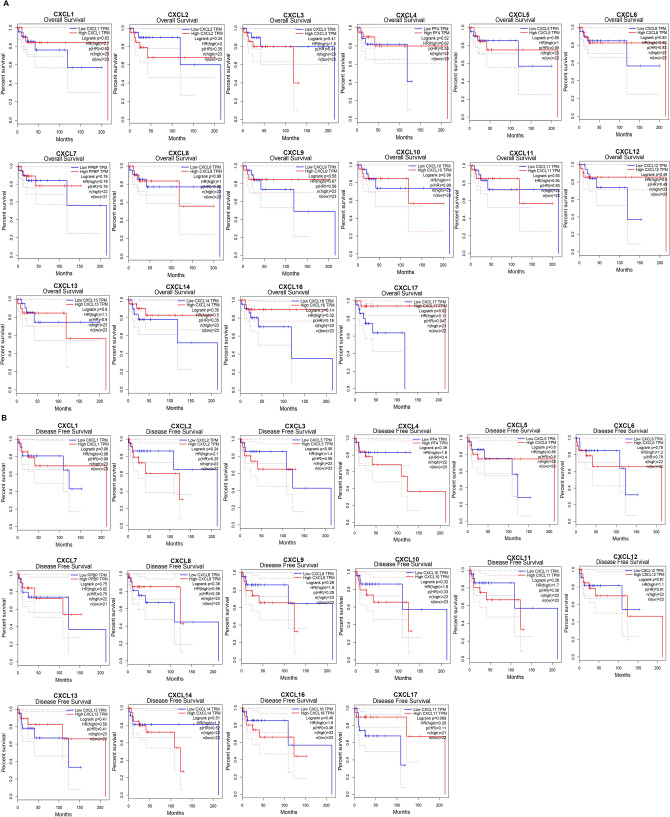
The prognostic value of mRNA level of CXCLs in DLBCL (GEPIA, http://gepia.cancer-pku.cn/). The prognostic value of mRNA level of CXCLs in DLBCL ((**A**) overall survival; (**B**) disease free survival).

We further investigated the role of CXCLs in the survival of patients with DLBCL. We used the GEPIA database to analyze the association of CXCL mRNA levels with survival in patients with DLBCL by using public datasets and used R (survminer package and survival package) to draw Kaplan–Meier curves. Kaplan–Meier curves and logarithmic rank tests showed that increased mRNA levels of CXCL9-14 and decreased CXCL4 and CXCL7-8 mRNA levels were not significantly associated with overall survival (OS) or disease-free survival (DFS) in all patients with DLBCL (P > 0.05). Higher CXCL17 mRNA levels were associated with prognosis in patients with DLBCL because of higher OS (P < 0.05) (Fig. 4A,B).

### Functional enrichment analysis of CXCL factors and adjacent genes in patients with DLBCL

**Figure 5:**
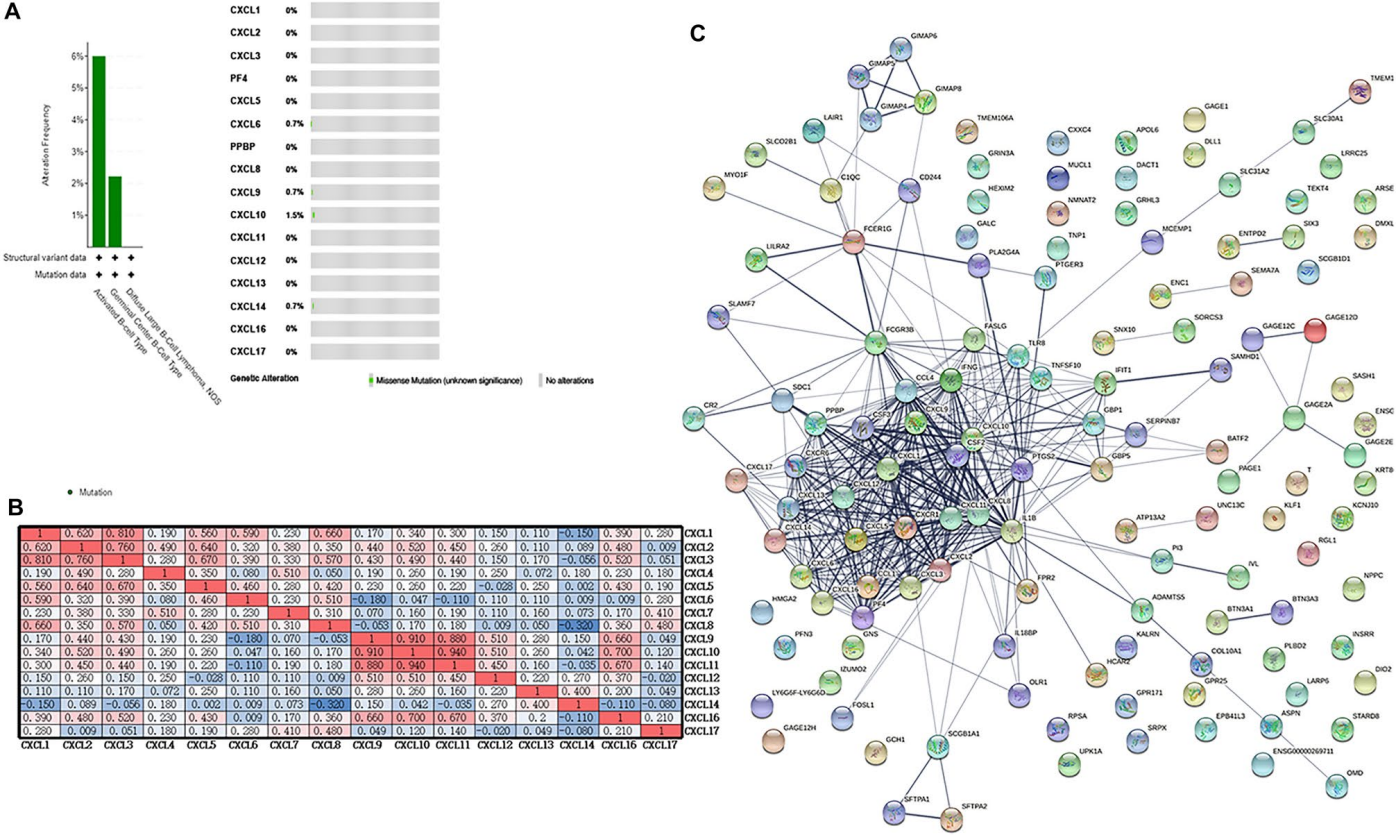
CXCL gene expression and mutation analysis in DLBCL. **(A)** CXCL gene expression and mutation analysis in DLBCL (cBioPortal, cBioPortal, https://www.cbioportal.org/). **(B)** Correlation between different CXCLs in DLBCL (EXCEL, version Microsoft Office Mondo 2016, https://office.microsoft.com/excel) (note: red indicates that the two parameters were positively correlated, and blue indicates that the two parameters were negatively correlated. The darker the color, the stronger the correlation). **(C)** Network for CXCLs and frequently altered neighboring genes (STRING, https://string-db.org/, version 11.5).

We used the cBioPortal online tool to analyze the changes in CXCLs. CXCLs were altered in 135 patients with DLBCL (3.6%). Changes were detected in approximately five samples (Fig. 5A). We also analyzed CXCL mRNA sequencing data by R (ggplot 2 package) and calculated the correlation between them and used EXCEL to complete the drawing (Fig. 5B). The correlations between CXCLs were shown in Fig. 5B. We also used STRING to construct a network of CXCLs and related adjacent genes. The results showed that the inflammatory cytokine IL16 and colony stimulating factor CSF3 were closely related to CXCLs (Fig. 5C).

We used R (ggplot2 package and clusterprofiler package) to statistically analyze and visualize the data, and predicted the functions of CXCLs alterations and the genes significantly related to CXCLs by analyzing Kyoto Encyclopedia of Genes and Genomes (KEGG).

**Figure 6:**
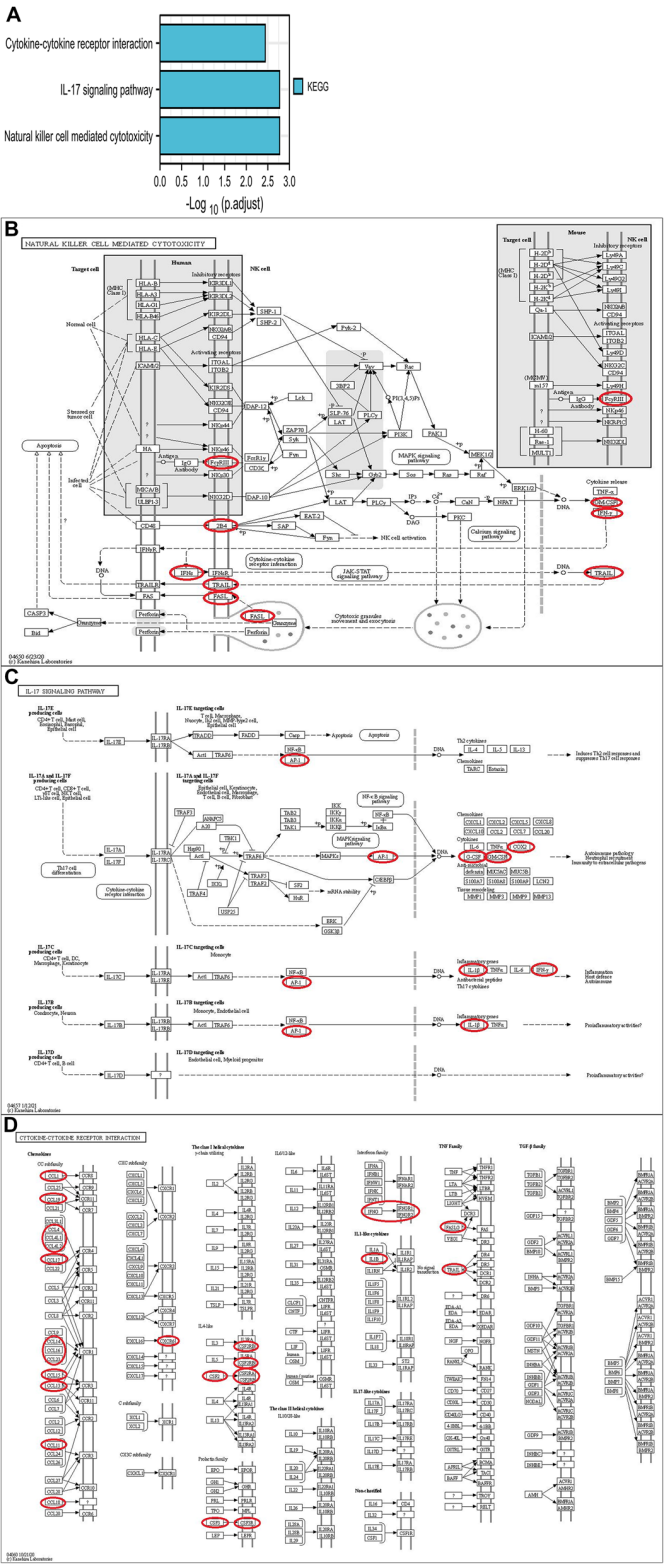
Kyoto Gene and Genome Encyclopedia (KEGG) pathways of CXCLs and genes significantly associated with CXCLs. **(A)** The functions of CXCLs and genes significantly associated with CXCLs were predicted by the analysis of Kyoto Encyclopedia of Genes and Genomes (KEGG); **(B)** natural killer cell mediated cytotoxicity; **(C)** IL-17 signaling pathway and **(D) **cytokine–cytokine receptor interaction regulated by the CXCLs alteration in DLBCL were shown.

KEGG analysis can identify the functional related pathways of CXCLs and neighboring genes. By KEGG analysis, we identified 10 pathways in reference to CXCLs function in DLBCL. Among these pathways, hsa04650 (natural killer cell-mediated cytotoxicity), hsa04657 (IL-17 signaling pathway) and hsa04060 (cytokine-cytokine receptor interaction) were involved in the tumorigenesis and pathogenesis of DLBCL (Fig. 6A–D).

### Correlation between CXCL expression and immune infiltration

**Figure 7:**
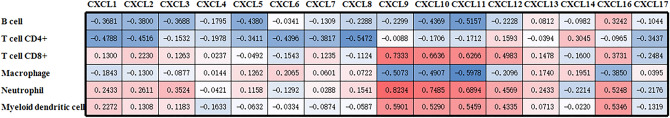
Correlation between CXCL expression and immune infiltration in DLBCL (EXCEL, version Microsoft Office Mondo 2016, https://office.microsoft.com/excel) (note: red indicates that the two parameters were positively correlated, and blue indicates that the two parameters were negatively correlated. The darker the color, the stronger the correlation).

Specifically, as shown in Fig. 7, CXCL1 expression was negatively correlated with CD4 + B cells and T cells. The expression of CXCL2 and CXCL5 had the same relationship with immune cells as CXCL1. CXCL3 expression was negatively correlated with CD4 + B cells and T cells; in addition, CXCL3 expression was positively correlated with neutrophils. The expression of CXCL6-8 was negatively correlated with CD4 + T cells. CXCL9-11 expressions were negatively correlated with B cells and macrophages; furthermore, CXCL9-11 expressions were appreciably positively correlated with CD8 + T cells, neutrophils and myeloid dendritic cells. CXCL12 expression was positively correlated with CD8 + T cells, neutrophils and myeloid dendritic cells. CXCL14 was positively correlated with CD4 + T cells but negatively correlated with CD8 + T cells. CXCL16 expression was positively correlated with the infiltration of several immune cell types, including B cells, CD8 + T cells, neutrophils and myeloid dendritic cells. Additionally, it was negatively correlated with CD4 + T cells. There was no marked infiltration with CXCL4 and CXCL17 in DLBCL.

## Discussion

CXCLs were dysregulated in a variety of cancers^32–34^. The role of CXCLs in the development, diagnosis and prognosis of several cancers has been partially established^35–37^. The data we obtained showed more insight into the bioinformatics analysis of DLBCL. This study is the first to investigate the expression and significance of different CXCL factors in DLBCL and is helpful to improve the diagnostic criteria and future treatment design on the basis of existing knowledge. It can also improve the diagnostic accuracy and prognosis of patients with DLBCL.

There are few studies on the expression of CXCLs in lymphoma except CXCL8 and CXCL11 in DLBCL^10,11^. Other CXCL factors have not been studied. The pathogenesis of CXCLs in DLBCL requires inspiration from other types of lymphoma and other cancers.

We have several hypotheses about the mechanisms of CXCLs and tumorigenesis. First, Epstein-Barr virus (EBV) positive DLBCL is a different entity. EBV can downregulate the expression of CXCL1 and transform human B lymphocytes by promoting the cell cycle and mitosis, inhibiting apoptosis, hindering host immune function and cytokine secretion to promote the production of tumors^38^. Taking CXCL1 as an example, we speculate that CXCLs can promote the production of EBV + DLBCL through the EBV infection-NF- κB pathway. Second, the expression of CXCLs can also be influenced by hypoxia inducible factor α (HIF-1α) in the tumor microenvironment. Hypoxia is a common feature of tumor microenvironment. Hypoxia leads to an increase in HIF-α through the NF-κB pathway, which leads to a change in CXCL expression levels. CXCLs facilitate angiogenesis and other pathways to promote tumor cells to obtain different proliferation and metastasis potentials^39^. Third, AP-1 family transcription factors regulate a variety of cellular activities in classical Hodgkin's lymphoma and anaplastic large cell lymphoma^40^. Whether CXCLs and their associated genes also promote tumor proliferation, inhibit apoptosis, and evade host immune responses through the AP-1 and NF-κB pathways remains unclear.

In this study, the Oncomine dataset and TCGA dataset showed no difference in CXCL1 expression levels between DLBCL patients and normal samples. ROC curves showed that CXCL1 had a low predictive value for DLBCL Using the Kaplan–Meier curve, which means that CXCL1 expression was not associated with the prognosis of patients with DLBCL.

Similarly, CXCL2 expression levels in DLBCL patients were not statistically significant in tumor tissues and normal samples. ROC curves showed that CXCL2 had a certain predictive value for DLBCL. CXCL2 expression was not associated with the prognosis of patients with DLBCL by Kaplan–Meier curve.

The expression levels of CXCL9-14 in tumor samples were higher than those in normal samples. Besides, CXCL2 and CXCL10-11 were related to the tumor stage. The expression of the CXCL family was not related to the prognosis except CXCL17. From this, we speculate that CXCL2 and CXCL17 may play an important role in the pathogenesis development of DLBCL.

Moreover, CXCLs and their closely related genes could induce and promote tumorigenesis in many ways. For example, natural killer cell-mediated cytotoxicity, the IL-17 signaling pathway and cytokine-cytokine receptor interactions are mentioned in the text. They might be related to apoptosis. Fas, IFN-γ, COX2, CSF, TRAIL and so on can induce or promote apoptosis (KEGG)^41–45^. We can further explore the expression of CXCLs and their mechanism of apoptosis in DLBCL.

The development of various tumors is highly related to the physiological state of the tumor microenvironment (TME)^46^. With the continuous understanding and deepening of the tumor immune microenvironment, it has great development potential in the prediction and guidance of immunotherapy^47^. According to the TIMER2.0 database, our results showed that the expression of CXCLs was positively correlated with the expression of tumor-infiltrating immune cells in the TME. CXCLs played an important role in TME. All components of the TME support tumor cells by producing growth factors, chemokines and so on, thus promoting tumor proliferation and metastasis.

## Conclusion

Overall, our study systematically analyzed the expression of CXCLs in DLBCL and its diagnostic and prognostic value, which provided a theoretical basis for further understanding the molecular biological and clinical characteristics of DLBCL. Clinical stages suggested that the high expression of CXCL2, CXCL10 and CXCL11 might leave a deep impression on the occurrence and development. The high expression of CXCL9-14 could also be used as an auxiliary molecular marker for its diagnosis. In addition, our results also suggest that transcribed CXCL17 may be a potential prognostic marker for improving survival and prognostic accuracy in patients with DLBCL.

## Data Availability

Materials and data from the study were available.
