# Author Correction: Comprehensive analysis of the expression and signifcance of CXCLs in human difuse large B-cell lymphoma

**DOI:** 10.1038/s41598-022-07486-9

**Published:** 2022-02-25

**Authors:** Xiaonan Zhou, Shizhu Guo, Yonghong Shi

**Affiliations:** 1 School of Basic Medical Sciences, Inner Mongolia Medical University, Hohhot, 010110 Inner Mongolia Autonomous Region China; 2 Department of Pathology, Affiliated Hospital of Inner Mongolia Medical University, Hohhot, Inner Mongolia Autonomous Region China

Correction to: *Scientific Reports* 10.1038/s41598-022-06877-2, published online 18 February 2022

The original version of this Article contained an error in Affiliation 1, which was incorrectly given as ‘School of Basic Medicine Sciences, Inner Mongolia Medical University, Hohhot, 010110, Inner Mongolia Autonomous Region, China’. The correct affiliation is listed below:

School of Basic Medical Sciences, Inner Mongolia Medical University, Hohhot, 010110, Inner Mongolia Autonomous Region, China

The original Article has been corrected.

